# The Impacts of Wind Speed Trends and 30-Year Variability in Relation to Hydroelectric Reservoir Inflows on Wind Power in the Pacific Northwest

**DOI:** 10.1371/journal.pone.0135730

**Published:** 2015-08-13

**Authors:** Benjamin D. Cross, Karen E. Kohfeld, Joseph Bailey, Andrew B. Cooper

**Affiliations:** School of Resource and Environmental Management, Simon Fraser University, 8888 University Drive, Burnaby, BC, Canada; Centro de Investigacion Cientifica y Educacion Superior de Ensenada, MEXICO

## Abstract

In hydroelectric dominated systems, the value and benefits of energy are higher during extended dry periods and lower during extended or extreme wet periods. By accounting for regional and temporal differences in the relationship between wind speed and reservoir inflow behavior during wind farm site selection, the benefits of energy diversification can be maximized. The goal of this work was to help maximize the value of wind power by quantifying the long-term (30-year) relationships between wind speed and streamflow behavior, using British Columbia (BC) and the Pacific Northwest (PNW) as a case study. Clean energy and self-sufficiency policies in British BC make the benefits of increased generation during low streamflow periods particularly large. Wind density (WD) estimates from a height of 10m (North American Regional Reanalysis, NARR) were correlated with cumulative usable inflows (CUI) for BC (collected from BC Hydro) for 1979–2010. The strongest WD-CUI correlations were found along the US coast (r ~0.55), whereas generally weaker correlations were found in northern regions, with negative correlations (r ~ -0.25) along BC’s North Coast. Furthermore, during the lowest inflow years, WD anomalies increased by up to 40% above average values for the North Coast. Seasonally, high flows during the spring freshet were coincident with widespread negative WD anomalies, with a similar but opposite pattern for low inflow winter months. These poorly or negatively correlated sites could have a moderating influence on climate related variability in provincial electricity supply, by producing greater than average generation in low inflow years and reduced generation in wet years. Wind speed and WD trends were also analyzed for all NARR grid locations, which showed statistically significant positive trends for most of the PNW and the largest increases along the Pacific Coast.

## Introduction

Global wind power generation is growing rapidly, with total investments of $99.5 billion and a record 49GW of newly installed capacity in 2014 alone [1]. While wind power offers many benefits compared to fossil fuels–including increased energy independence, and lower operational costs, emissions, and impacts from resource extraction–its intermittent nature is a large challenge to effective integration with existing electricity grids.

One of the most promising solutions to the issue of intermittency is energy storage in existing hydroelectric reservoirs [2–6]. Large hydroelectric dams have the benefits of large storage capacity, being rapidly dispatchable, and tending to exist in energy limited, rather than capacity limited, energy systems that can benefit from additional energy from intermittent sources [7].

Many studies have addressed the challenges of integrating wind and hydro power at the operational level, such as real-time voltage fluctuations and next-hour forecasting [8–11], and the benefits of matching diurnal electricity demand and wind speed profiles [12–15]. However, the importance of the relative long-term, multi-decadal variability in wind speeds and hydroelectric reservoir inflows has not been explored. Like wind power, hydropower is also susceptible to climatic variability in the form of variations in reservoir inflows [16]. In extreme or extended cases, low inflows can lead to decreased energy supply, increased prices, or energy insecurity [16,17]. Similarly, high inflow periods can result in low or even negative prices, exceeding environmental flow limitations, spillage, and curtailment of other energy sources [18,19]. Accounting for the relative variability between hydropower and wind power could lead to the selection of wind farm locations that provide a greater economic value to the energy system as a whole due to more beneficial timing.

For example, wind farms with above-average generation during low inflow periods would have greater value than otherwise similar sites where most generation occurs during periods of high inflows and reservoir levels when electricity prices are typically lower. Similarly, sites with below-normal generation during high inflow periods help to maintain system flexibility, thereby reducing the need to spill from overfilled reservoirs and pay wind farms to curtail production, as occurred in Washington State during the freshet of 2011 [18].

The Canadian province of British Columbia (BC), and the broader Pacific Northwest (PNW) region, were chosen to explore the spatial variability of the 30-year co-variability in wind speeds and hydroelectric reservoir inflows and to identify regions with beneficial and detrimental wind speed timing. The potential benefits of wind and hydroelectric resources with poorly-correlated temporal behavior are particularly strong in BC, where hydropower accounts for 95% of all electricity generation [20] and the BC *Clean Energy Act* (2010) [21] has required that at least 93% of all generation come from clean or renewable sources. The *Clean Energy Act*’s self-sufficiency requirement also requires that in-province generation be able to meet all domestic demand by 2016, based on an average water year. However, unlike many other hydroelectric dominated systems, BC is capacity limited under most conditions, making additional generation during periods of high demand more important, but reducing the utility of additional energy except during extended drought periods [22].

Given a projected 20% increase in BC population over the next two decades, energy forecasts for BC suggest a strong likelihood that energy demand will also increase [23]. Continuing to rely on hydropower in BC will likely require the development of substantial new hydroelectric resources due to hydroelectricity’s interannual variability and the need to ensure self-sufficiency, which requires a much greater generation capacity than is needed in many years. Hydroelectric overdevelopment would also have large environmental and social costs due to flooding, road and transmission line construction, and streamflow changes [24]. Instead, the economic and environmental costs of constructing a reliable energy system could be reduced by selecting alternative sources that have consistent or increased production in low water periods. Wind power is a potentially strong option for diversifying BC’s electricity mix, with the lowest levelized cost of all sources in BC Hydro’s 2008 Clean Energy Call and making up nearly half of the resulting firm energy agreements [25], while still achieving BC's goals of low-carbon energy intensity.

As a case study, the PNW also has the advantage of including a wide geographical range that encompasses several climatic regions and spans an international border. The region includes coastal and inland sites, a large latitudinal range, and multiple mountain ranges. Long–term wind behavior is likely to differ between these regions, and a heterogeneous wind resource increases the likelihood that some areas will exhibit beneficial multi-decadal wind timing relative to hydroelectric inflows. The strong linkages between BC and west coast United States energy systems mean there are also implications for international energy trading and market prices, particularly for the large installed wind power capacity in Oregon and Washington States.

Several wind speed studies have included or been conducted in the PNW, but the results have been inconsistent and their geographic extent in BC is very limited [26–36]. Griffin et al. (2010) [37] reconciled some of these inconsistencies by studying a larger geographic area, finding small negative trends at inland sites, similar to the general stilling trends observed across much of the continental US [31], and cyclic behaviour with no significant trends at coastal sites. While these studies provide valuable information for certain locations, their applicability to the whole region is severely limited by the exclusive use of meteorological station data and a focus on the coastal and southern portions of the PNW.

The objectives of this study are three-fold. First, we use the North American Regional Reanalysis (NARR) data [38] to produce an analysis of long term (30-year) wind speed behaviour with better spatial and temporal extent than is available using meteorological station data alone. Second, we analyze the relationship between the 30-year variability (interannual and seasonal) in PNW wind speeds and inflows into BC’s hydroelectric reservoirs, considering both interannual correlations and wind speed anomalies in the highest and lowest inflow years and seasons. Finally, we consider other regions where the proposed methodology could be applied to improve wind farm site selection based on energy system characteristics and the climatic causes of regional variations in wind speed-inflow relationships.

## Data and Methods

**Fig 1:**
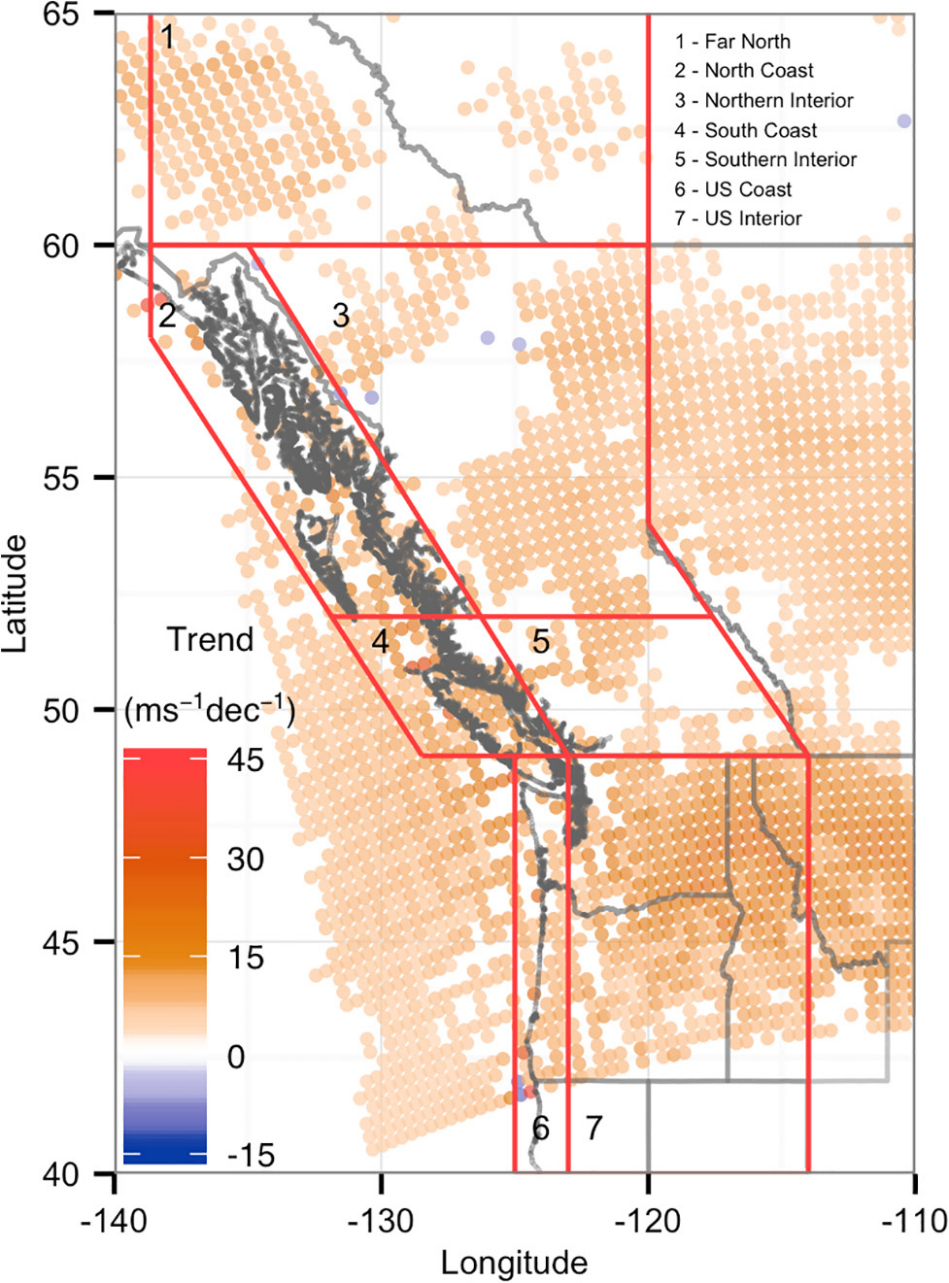
Linear trends [m s^-1^ (10 yr)^-1^] in average monthly near-surface (10m) wind speeds from 1979 to 2010. Results are shown are all NARR grid locations in the Pacific Northwest with significant (P < 0.1) trends.

Although the study area included a broad definition of the PNW (40° to 65°N and 110° to 135°W) to provide a more effective investigation of latitudinal and geographic patterns in wind behavior, the study focused on the province of British Columbia, the states of Washington and Oregon, and the nearshore regions where offshore wind farms would be feasible. The study area was separated into 7 distinct regions based on political boundaries, proximity to the coast, and the divide between northern and southern British Columbia (Fig 1). Regional averages were calculated using the inverse variance weighting method to give greater priority to more robust anomalies and correlations. Average results were also calculated for BC’s four existing wind farms and for the 164 potential onshore and offshore wind farm locations identified in BC Hydro’s 2010 Resource Option Report [39].

All wind speed data were obtained from the North American Regional Reanalysis (NARR) dataset [38], produced by the National Center for Atmospheric Research (NCAR) and the National Centers for Environmental Prediction (NCEP). The NARR project is an extension of the NCEP-DOE Global Reanalysis (GR) that uses the high resolution NCEP Eta Model and its Data Assimilation System, the Noah land surface model, and several additional datasets compared to those of the GR to produce a higher resolution and more accurate reanalysis product for North and Central America [38]. This process assimilates observations of many meteorological variables, including wind speed, temperature, precipitation, and pressure, onto a three-dimensional grid system with 45 vertical layers and a horizontal spacing of approximately 32 km [38]. As the proposed methodology relies on the relative variability of wind speeds, and not absolute wind speeds, 10m winds were used rather than hub height (i.e., the height of the centre of the turbine rotor relative to the ground surface) to maximize the influence on the NARR data of the station data that do exist in BC. Three-hourly wind speeds were extracted from the NARR dataset from 1979–2010. To better represent wind power potential, monthly wind density (WD) totals were then calculated by cubing each value and summing for each month, season, and year.

Reservoir inflow data for BC were obtained from BC Hydro in the form of monthly combined usable inflows (CUI, GWh) for 1979 to 2010, which were summed to produce seasonal and annual totals (S1 Table). CUI is a provincial summary of energy generating potential of all inflows into the major hydroelectric reservoirs, which are weighted to account for each dam’s individual long-term average energy conversion rate, and includes water flow through non-power release facilities. CUI was chosen over other streamflow and precipitation measures because of its direct relationship to total potential hydroelectric generation and therefore its ability to identify when the hydroelectric system is most under stress due to extended wet or dry periods. However, it should be noted that CUI does not account for the mitigating effects of energy storage on interannual energy supply. For example, BC Hydro has two reservoirs with multi-year storage capabilities, while smaller systems and run-of-river facilities may have no or very limited storage capability.

For each NARR grid location in the study area, linear trends were calculated for monthly mean winds and WD totals. While the assumptions of ordinary least squares regression (OLS) are not likely to be met for climate trend analysis, due to the cyclic, autocorrelated nature and non-normal distribution of wind speed and wind density data [32,40], OLS was used to allow for better comparisons with previous wind trend studies, which used OLS almost exclusively [40].

Three sets of analyses were performed to understand the interannual and seasonal relationships between WD and CUI. First, interannual correlations were calculated between annual WD and CUI totals for each NARR grid location using Pearson correlation coefficients. Although the assumption of a linear relationship is unlikely to be met, Pearson was chosen because other nonparametric tests, such as Spearman’s rank correlation, reduce the sensitivity to extreme values, which were our primary interest.

Second, to determine whether WDs during the lowest inflow years were significantly different from those in other years, a non-parametric Mann-Whitney U (aka Rank Sum) test was used to compare WD totals for the lowest CUI years and WD totals in all remaining years. The two populations were considered distinct if the sum of the ranks of each population were statistically different (p < 0.1). This test was performed for the three and five lowest inflow years to test if the results were robust across low inflow years in general, or were rather a product of the particular years in question (Table 1). The same analysis was repeated for the highest three and five inflow years. The purpose of this analysis was to identify broad regions of interest in which the patterns of wind speed behavior are different from CUI during extreme years. Thus, we chose a significance level of p < 0.1, so as to avoid eliminating sites that may still have beneficial wind timing. For the purpose of identifying these general patterns of interest, we were willing to accept a 10% chance of a type 1 error for any single location.

**Table 1:** Statistics for British Columbia’s annual cumulative usable inflows (CUI).

Rank	Year	Percent of Median Annual CUI Total(1979–2010)
**High Flow Years**		
1	1996	120.4%
2	1995	119.9%
3	2006	112.3%
4	1998	112.1%
5	2010	110.8%
**Low Flow Years**		
28	1984	89.8%
29	1992	89.4%
30	2008	87.3%
31	1979	84.5%
32	2009	84.0%
**Standard Deviation**		9.4%

Finally, to determine if wind density totals for the highest and lowest inflow seasons were statistically different from the rest of the year, the same analysis was performed for the winter (December-March) and freshet (May-July) seasons, testing the WD totals in those months against all remaining months of the year. Annual CUI and WD totals were based on the October through September water year, while seasonal analyses included the freshet period (May through July) when inflows are highest in the PNW, and winter (December through March) when inflows are lowest.

## Results

### Wind Speed and Wind Density Trends

The analysis of 10m NARR monthly average wind speeds showed significant positive trends (p < 0.1) for most of the PNW (Fig 1). However, the Far North, Northern Interior, and Southern Interior regions showed less consistent results, with more sites exhibiting non-significant trends.

The inverse variance weighted mean trend was similar for most regions (Table 2), however there was greater variation in the range of trends within each region. The North, South, and US Coast regions contained local maxima of 0.480 to 0.561 m/s/decade (10.1 to 12.7%/decade). However, the US and North Coast regions also contained locations with some of the strongest negative trends, as low as -0.184 m/s/decade (-3.30%/decade). In contrast, the Far North and the three Interior regions had relatively small differences between the maximum and minimum trends, with trend ranges between 0.260 to 0.342 m/s/decade (6.05 to 7.42%/decade), compared to the much larger trend range of 0.744 m/s/decade (16.0%/decade) for the US Coast.

**Table 2:** Summary of regional results for the Pacific Northwest.

Region	Wind Speed Trend (m s^-1^ (10 yr)^-1^) (% (10 yr)^-1^)	WD Trend (m^3^ s^-3^ (10 yr)^-1^) (% (10 yr)^-1^)	WD-CUI Correlation
Far North	0.0668 (1.79%)	2.56x10^3^ (5.47%)	0.0326
North Coast	0.0598 (1.24%)	1.14x10^4^ (6.38%)	0.107
Northern Interior	0.0698 (1.95%)	4.05x10^3^ (7.19%)	0.185
South Coast	0.0620 (1.16%)	2.95x10^3^ (12.1%)	0.151
Southern Interior	0.0629 (1.83%)	2.97x10^3^ (6.34%)	0.202
US Coast	0.121 (1.86%)	6.41x10^3^ (10.5%)	0.228
US Interior	0.0614 (1.84%)	4.79x10^3^ (9.57%)	0.184

Table includes inverse variance weighted averages of near-surface (10m) mean monthly wind speeds and wind density totals, and inverse variance weighted correlations between total annual wind density and CUI. See Fig 1 for region definitions.

**Fig 2:**
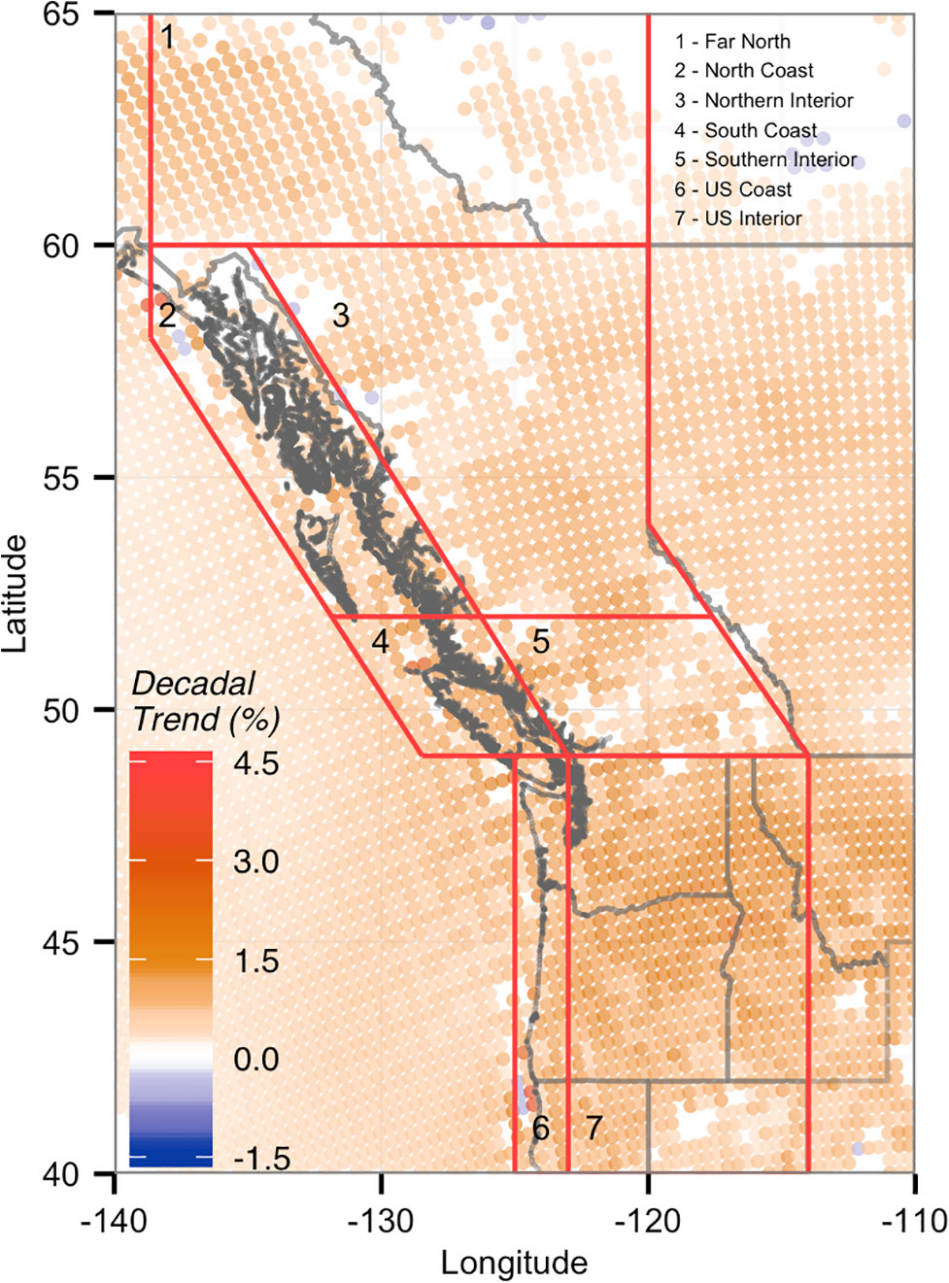
Linear trends [% (10 yr)^-1^] in total monthly near-surface (10m) wind density from 1979 to 2010. Results are shown are all NARR grid locations in the Pacific Northwest with significant (P < 0.1) trends.

Wind density trends exhibited a similar pattern to the monthly mean wind speeds, with large increases for most of the PNW (Fig 2). The strongest trends were seen in the South Coast, US Coast, and the US Interior (Table 2), with many sites experiencing increases greater than 10%/decade, and some increases of up to 33%/decade. All of the northern regions, and the Southern Interior, generally exhibited smaller positive trends and contained more sites with non-significant trends (Fig 2). Only 14 NARR grid locations exhibited significant negative wind density trends (P < 0.1), mostly isolated to the northern Alaskan panhandle and the Great Slave Lake region of the Northwest Territories.

Mean wind speed and density trends at BC’s existing and potential wind farm sites were all positive and larger than their respective regional averages, except for the South Coast where the regional average wind speed trend was larger (Table 3). The largest WD increases, up to 10%/decade, were found in the South Coast and Northern Interior, the two regions which contain all of BC’s existing wind farms.

**Table 3:** Summary of regionally averaged results for all potential and existing wind farm sites in British Columbia.

Potential Wind Farm Locations by Region	Wind Speed Trend (m s^-1^ (10 yr)^-1^) (% (10 yr)^-1^)	WD Trend(m^3^ s^-3^ (10 yr)^-1^) (% (10 yr)^-1^)	WD-CUI Correlation
Far North	0.109 (6.30%)	5.64 x10^3^ (6.09%)	0.0957
North Coast	0.108 (4.95%)	3.03 x10^4^ (7.81%)	-0.0158
Northern Interior	0.0887 (6.23%)	1.20 x10^4^ (8.17%)	0.216
South Coast	0.0211 (0.822%)	3.42 x10^4^ (10.0%)	0.179
Southern Interior	0.0786 (6.15%)	7.60 x10^3^ (7.20%)	0.246
Existing (Northern Interior and South Coast)	0.0593 (4.59%)	2.09 x10^4^ (9.52%)	0.177

Table includes inverse variance weighted averages of near-surface (10m) mean monthly wind speeds and wind density totals, and inverse variance weighted correlations between total annual wind density and CUI. See Fig 1 for region definitions.

### Interannual Wind Density-CUI Correlation

**Fig 3:**
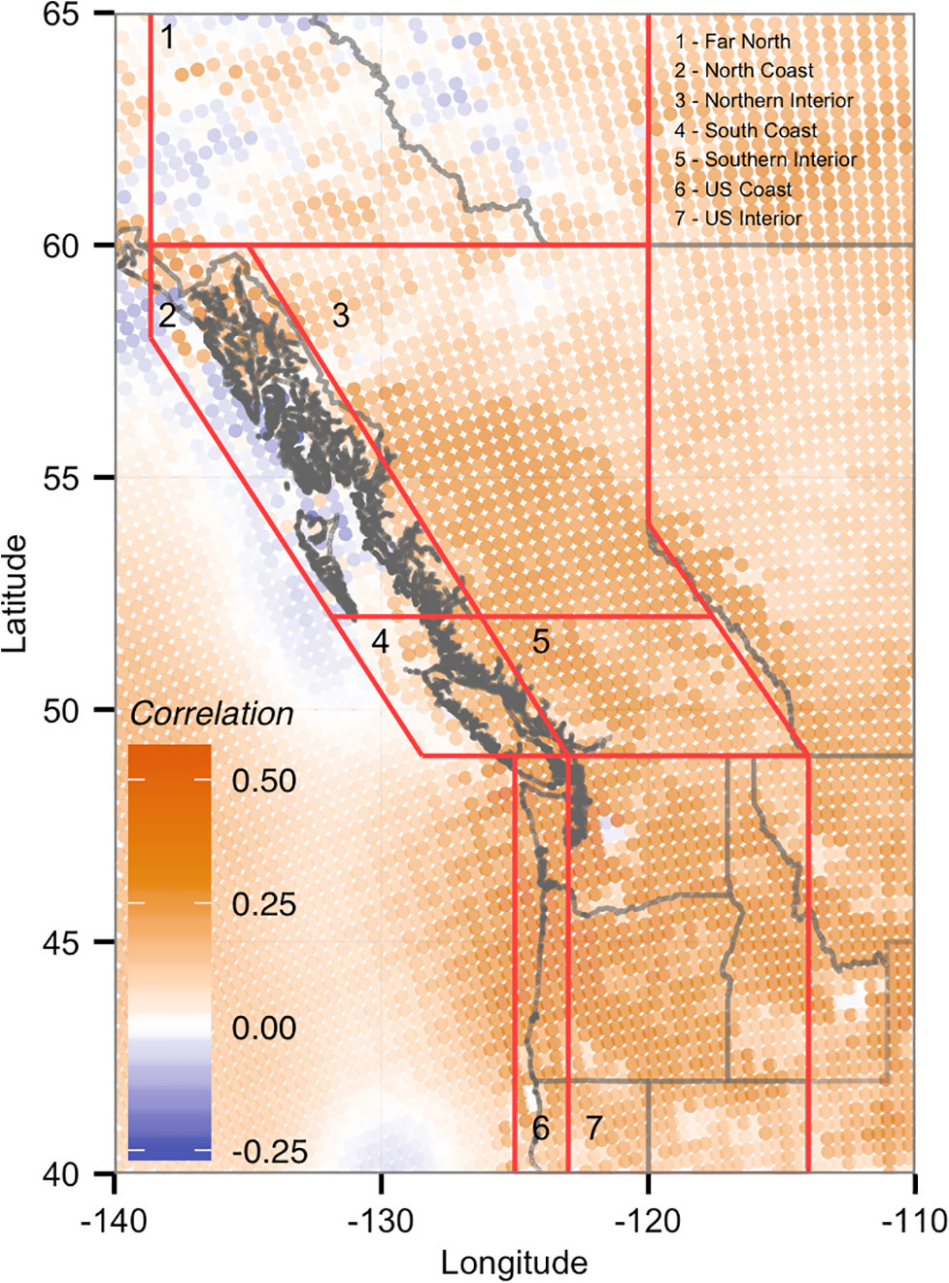
Correlations between British Columbia’s annual CUI and surface (10 m) wind density totals from 1979 to 2010. All annual totals are based on the water year of October through September, referenced by the calendar year containing October to December. Results are shown for each NARR grid location in the Pacific Northwest.

Most of the PNW exhibited non-significant (p > 0.1) positive correlations between annual CUI and WD totals (Fig 3). However, all regions contained individual sites with significant positive correlations. The strongest correlations were found in the Far North, South Coast, and US Coast and Interior, with Pearson’s r values up to 0.556.

Most regions had at least one location with a negative WD-CUI correlation, but even the strongest negative correlations, located in the North Coast and Far North regions, were not significant, with minimum values as low as -0.281.

All of the potential wind farm sites had similar WD-CUI correlations to their respective regional averages (Table 3). The potential wind farm sites on the North Coast had the greatest difference from the regional average, with a mean WD-CUI correlation of -0.0158, compared to 0.107 for the region.

### Wind Density Anomalies in Low Inflow Years

**Fig 4:**
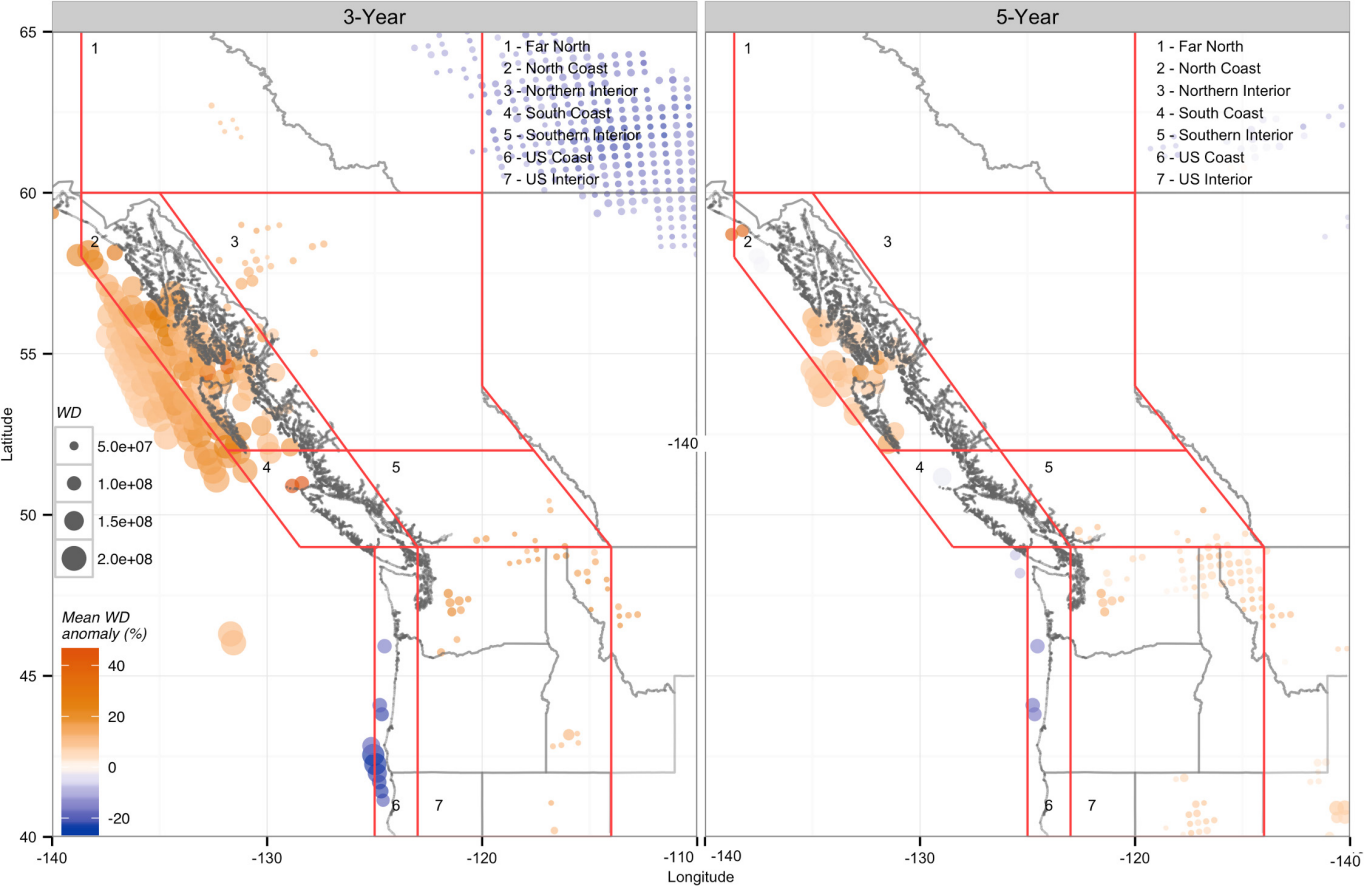
Average anomalies in annual near-surface (10m) wind density totals in the three and five lowest CUI years from 1979 to 2010. Colour indicates significant positive (orange) or negative (blue) correlations, and dot size indicates relative wind density totals over the entire NARR data record. All annual totals are based on the water year of October through September, referenced by the calendar year containing October to December. Only locations that show statistically significant (P < 0.1) differences from annual wind density totals over the entire NARR data record (1979–2010) are shown. The five lowest CUI years in ascending order are 2009, 1979, 2008, 1992, and 1984.

Wind density anomalies (the difference between the average WD total for the period in question compared to the average annual WD for 1979–2010) varied substantially from region to region for the lowest inflow years (Fig 4). The North Coast had a large number of positive WD anomalies in both the three and five year cases, with annual WDs increasing by an average of approximately 15% and 10%, respectively, compared to average years. The US Interior showed similar results to the North Coast, with many positive anomalies and average increases of up to 14.7% in the three driest years. In contrast, the US Coast was the only region to exhibit negative anomalies in both the three and five lowest inflow years, with average declines of 21.3% and 15.6%, respectively, during low inflow years.

The other regions (Far North, Northern Interior, and South Coast) contained sites with significant anomalies in the three lowest inflow years, but all exhibited substantially different behavior when considering the five lowest inflow years. The Far North had many weak negative anomalies in the three lowest inflow years, but no sites exhibited significant anomalies in the five-year case (Table 4). Like the Far North, the Northern Interior only exhibited significant anomalies in the three-year case; however, these anomalies were all positive, with an average increase of 11.0%. The South Coast had few, but strongly positive anomalies in the three-year case, resulting in the largest regional average increase of 25.9%. However, in the five-year case the anomalies at these sites were no longer significant, causing the regional average to become weakly negative.

**Table 4:** Regionally averaged anomalies in annual near-surface (10m) wind density totals in the three and five lowest and highest CUI years.

	Low Inflow Years		High Inflow Years	
Region (Number of grid locations)	Mean 3-yr WD Anomaly (# Sites with Significant Anomalies)	Mean 5-yr WD Anomaly (# Sites with Significant Anomalies)	Mean 3-yr WD Anomaly (# Sites with Significant Anomalies)	Mean 5-yr WD Anomaly (# Sites with Significant Anomalies)
Far North (767)	-0.966% (34)	15.8% (2)	69.2% (9)	94.1% (3)
North Coast (220)	14.7% (87)	8.55% (37)	39.6% (6)	46.3% (1)
Northern Interior (415)	11.2% (14)	NA (0)	43.9% (73)	48.6% (33)
South Coast (121)	25.9% (5)	-3.69% (1)	39.1% (3)	48.2% (10)
Southern Interior (199)	15.1% (7)	7.19% (12)	72.2% (4)	62.5% (47)
US Coast (151)	-21.3% (8)	-15.6% (3)	46.5% (56)	39.6% (88)
US Interior (685)	14.7% (31)	5.95% (69)	53.0% (277)	53.5% (379)

Anomalies are presented as the regional average percent change in wind density for NARR grid locations with significant anomalies compared with the average wind density for 1979–2010. Numbers in parentheses represent the number of NARR grid locations with statistically significant anomalies in each region. All annual totals are based on the water year of October through September, referenced by the calendar year containing October to December.

Anomaly magnitudes and geographic patterns of significance were similar for both the three and five year low inflow cases, but the number of locations with significant anomalies was much higher for the three lowest CUI years compared to the five lowest years for most regions. However, the US Interior had more than double the number of significant anomalies for the five lowest inflow years compared to the three lowest inflow years (Table 4).

### Wind Density Anomalies in High Inflow Years

**Fig 5:**
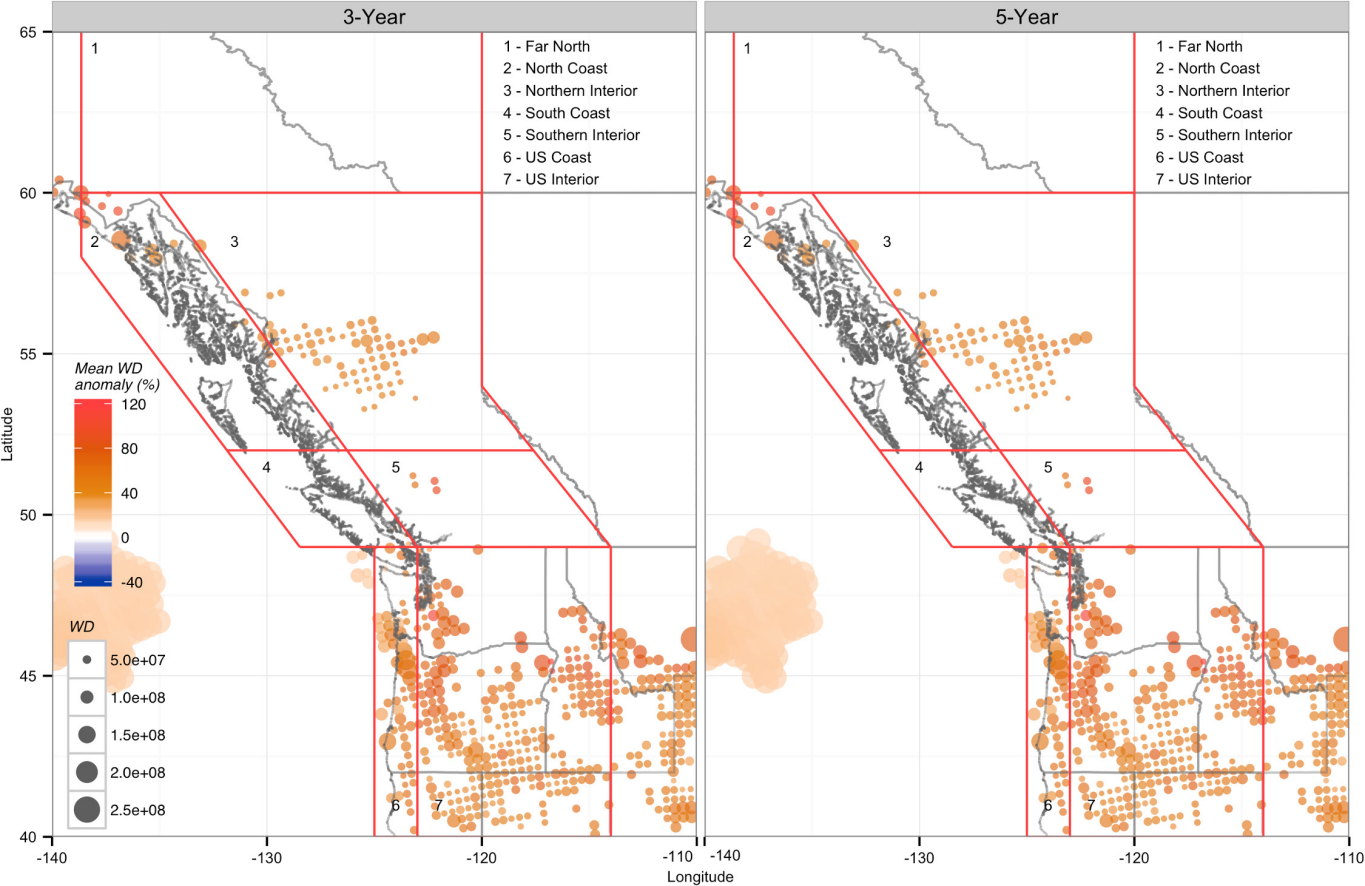
Average anomalies in annual near-surface (10m) wind density totals in the three and five highest CUI years. Colour indicates significant negative correlations, and dot size indicates relative wind density totals over the entire NARR data record. All annual totals are based on the water year of October through September, referenced by the calendar year containing October to December. Only locations that show statistically significant (P < 0.1) differences from annual wind density totals over the entire NARR data record (1979–2010) are shown. The five highest CUI years in descending order are 1996, 1995, 2006, 1998, and 2010.

Wind density anomalies for the highest inflow years were more homogeneous than for the lowest inflow years (Fig 5), with positive anomalies for all regions included in the study (Table 4). The largest anomalies were found in the Far North, North Coast, and Southern Interior, where WD increased as much as 85.5% compared to average years during the three highest inflow years. However, the anomalies in these regions were relatively sparse compared to the US Interior, which had the most significant anomalies and many of the highest magnitudes, with an average WD increase of close to 53.0% (Table 4).

When comparing the three and five-year high inflow cases, the locations with significant wind density anomalies for the five highest years were shifted slightly southward when compared with those for the three highest years. The number of sites with significant anomalies in the five-year case declined for the North Coast and Northern Interior, but increased for all other regions (Table 4). Many of the anomalies located in central BC in the three-year highest inflow case appear to shift to the south and became aligned with the Coastal and Rocky Mountain ranges when the five highest inflow years are considered (Fig 5). The US results are less varied between the two cases, with widespread positive anomalies for both the three and five year scenarios.

### Seasonal Variability

**Fig 6:**
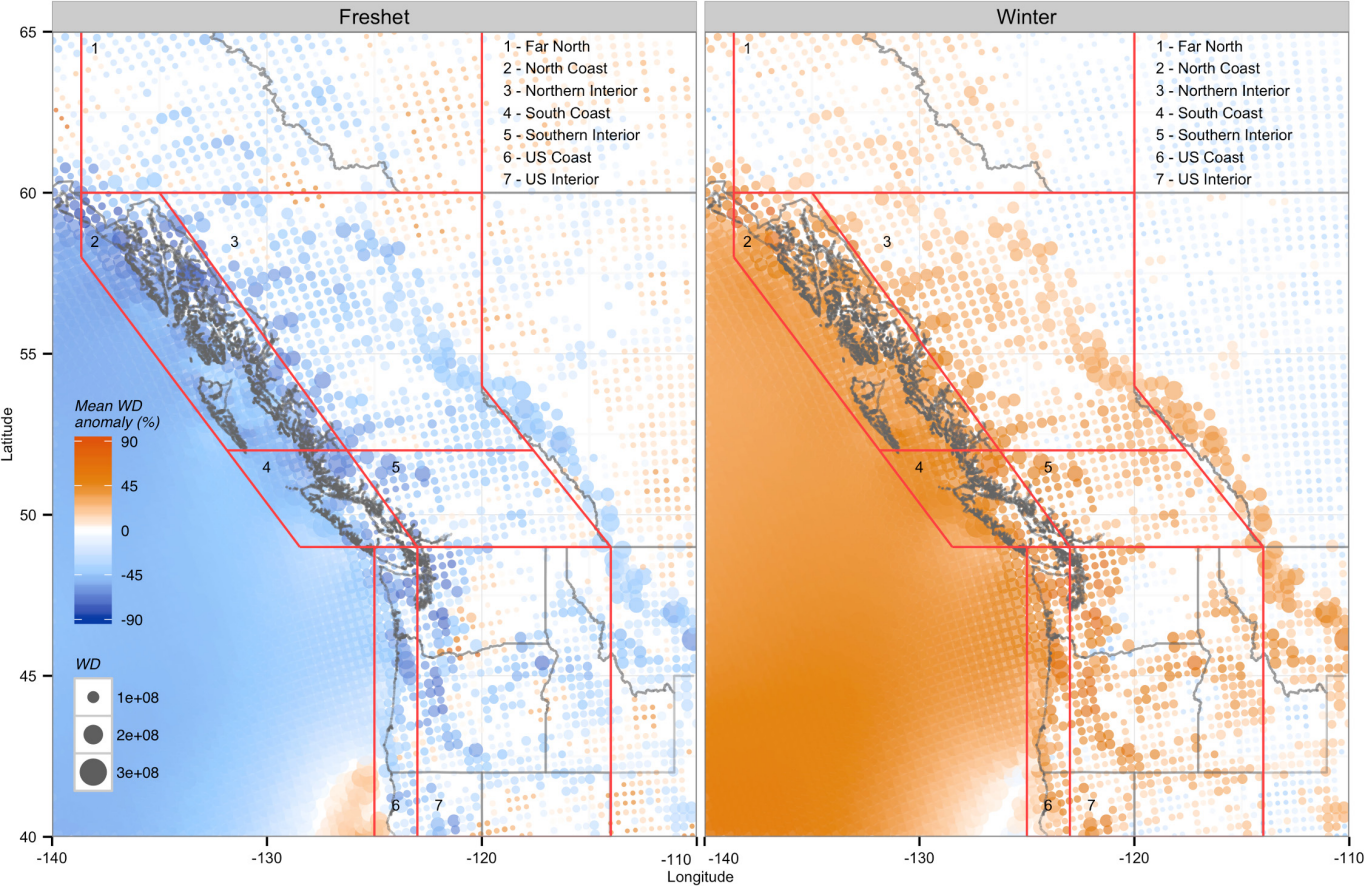
Average anomalies in monthly near-surface (10m) wind density totals in the winter (Dec–March) and freshet (May–July) seasons. Colour indicates significant negative correlations, and dot size indicates relative wind density totals over the entire NARR data record. Only locations that show statistically significant (P < 0.1) differences from monthly wind density totals over the entire NARR data record (1979–2010) are shown.

Almost all sites exhibited significant WD anomalies in the winter and freshet seasons (Fig 6), compared to the rest of the year. The relative magnitudes and geographic patterns of winter and freshet anomalies were very similar but opposite in sign, with positive WD anomalies west of the Rocky Mountains in winter and negative WD anomalies during the freshet. The strongest positive winter anomalies and negative freshet anomalies were found in the three coastal regions (Table 5), with maximum values up to 88.4% in the North Coast. Similarly, the North Coast also had the strongest freshet anomalies, where WD decreased by as much as 85.3% compared to an average month. However, all other regions also had relatively large anomalies, ranging from -67.0% in the Southern Interior to -74.1% for the US Interior.

**Table 5:** Regionally averaged anomalies in monthly near-surface (10m) wind density totals for the winter (Dec—March) and freshet (May—July) seasons.

Region (Number of grid locations)	Regionally averaged Winter WD Anomaly (# NARR Sites with Significant Anomalies)	Regionally averaged Freshet WD Anomaly (# NARR Sites with Significant Anomalies)
Far North (767)	3.69% (627)	-16.0% (661)
North Coast (220)	39.6% (220)	-54.2% (220)
Northern Interior (415)	19.0% (370)	-30.7% (380)
South Coast (121)	42.5% (121)	-48.0% (121)
Southern Interior (199)	23.4% (173)	-28.0% (173)
US Coast (151)	36.1% (145)	-37.7% (138)
US Interior (685)	18.7% (548)	-19.6% (526)

Anomalies are presented as the regional average percent change in wind density for NARR grid locations with significant anomalies compared to an average month. The number of NARR grid locations that exhibit significant anomalies in each case are shown in parentheses.

## Discussion

### Wind Speed and Wind Density Trends

For most regions the NARR wind speed trends (Fig 1) were inconsistent with the declining or cyclic behavior seen in recent studies of surface wind observations in southern BC [26,33,34,37], and the continental United States [31,36,40]. However, the positive trends do match well with other studies of reanalysis data in the American PNW [31,35], and with studies of surface observations at higher latitudes in Alaska, the Canadian Arctic, and the Antarctic [41,42]. Trends found in PNW station data have also typically been smaller and less geographically consistent than the widespread stilling seen for much of the rest of North America [31,40], and therefore the positive trends seen in the NARR data may not be entirely inconsistent with the observational data.

The increasing wind speeds at mid- and high-latitudes seen in the NARR data are consistent with many of the predicted effects of recent climate warming. Studies have suggested that the zone of tropical convection has been widening in recent decades [43], which has been accompanied by poleward shifts in the zonal mean midlatitude westerlies, tropospheric jet [44,45], and Pacific storm tracks [46,47]. Holt and Wang (2012) [35] found that increasing wind speeds in Washington and Oregon were almost exclusively due to a strengthening of the zonal, westerly wind component. This demonstrates that the strengthening and poleward shift in the storm tracks and midlatitude cyclones could also be contributing to the positive wind trends found for most of the PNW.

If the positive wind speed and wind density trends observed here continue, this would bode well for the potential of wind energy in BC and the PNW. In particular, there is already one industrial scale wind power development, and many proposed sites, along BC’s Pacific Coast where the largest positive trends were observed. However, this result represents a historical analysis covering only 30 years, which may not capture the potential influence of multi-decadal climate oscillations on wind speed behaviour. For example, using 50- and 60- year time series, previous studies have demonstrated substantial inter-decadal variability in the intensity of winter storm tracks and winter wind intensity along the Pacific coast [48–50]. Furthermore, Bylhouwer et al. (2013) [50] observed that wind speed intensity trends in the 60-year NCEP time series were less significant compared to NARR’s shorter record, suggesting that these wind speed trends should be interpreted with care.

### Climatic Implications of Wind Density–Inflow Interactions

The spatial variability in the interannual WD-CUI relationship is likely due to different regional responses to large-scale climate cycles, such as the El-Nino Southern Oscillation (ENSO), the Pacific Decadal Oscillation (PDO), and related shifts in storm track location.

Whereas WD is determined by wind speed behaviour at a specific location, CUI is a weighted aggregate of inflows into BC’s hydroelectric reservoirs, and is therefore dependent on the combined behaviour of all contributing watersheds. Approximately 81% of BC Hydro’s annual hydroelectric generation comes from the Peace and Columbia basins [20], located in the Northern Interior and Southern Interior regions, respectively, which means that CUI variability is largely determined by streamflow behaviour in these regions.

Both ENSO and PDO have been shown to be important influences on wind speed, precipitation, and streamflow in the PNW [33,51,52], but their effects vary across the region. Warm phases (positive indices) of both ENSO (El Nino) and PDO are generally associated with reduced precipitation, streamflow, and storminess in the PNW, while cold phases (negative indices) are associated with colder, wetter, and stormier weather [52–54]. However, ENSO is positively correlated with streamflow in the interior regions (such that warm phases are linked with increased precipitation) and negatively correlated on the coast [52]. The influence of warm phases of the PDO on low streamflow events is stronger on the coast than inland, and the effect decreases moving from south to north [52].

Wind speeds in the PNW are also influenced by ENSO and PDO [26,27,33], but location also plays a large role in determining both average and extreme winds [37]. Peak winds were also found to be higher during cold (La Nina) ENSO phases, with small decreases during warm phases [26,27]. Positive (warm) PDO phases are also associated with a high pressure system over western North America, lower air pressure gradients, and therefore less storm activity and lower mean wind speeds in the PNW. Likewise, negative (cold) PDO phases are associated with stronger pressure gradients and higher mean wind speeds [33]. However, Griffin et al. (2010) [37] showed that the strongest predictor of wind speed behaviour is site location, with coastal sites following an eight- to nine-year cyclic pattern, rather than directly following ENSO or PDO.

The WD-CUI relationship at a given location is therefore determined by how similar the wind speed behaviour at that site is to the precipitation and streamflow behaviour in the watersheds of the major dam reservoirs, located in the Northern and Southern Interior regions. For example, low inflow years in the interior regions are most likely to occur during negative (cold) ENSO phases, which bring strong winds to coastal areas and weaker winds to interior areas, as seen in Figs 3 and 4. More generally, wind speed and precipitation are often closely linked, particularly during stormy periods, and therefore locations close to the major hydroelectric reservoirs are more likely to have positive WD-CUI correlations.

Locations whose wind speed behaviour is influenced by factors other than large-scale climate cycles, for example due to location (e.g. on the coast [37]) or local topography, are also more likely to have low or negative WD-CUI correlations. Wind speed variability at these locations is less likely to be similar to reservoir inflow variability, which is largely controlled by regional circulation patterns associated with different phases of ENSO and PDO.

### Implications of Wind Density–Inflow Interactions for Energy Generation

The dominance of hydroelectricity in the PNW, combined with natural variation in precipitation and streamflow, result in large seasonal and interannual fluctuations in potential generation, which can place the electricity system under stress during extreme high and low inflow periods. The value of energy, capacity, and system flexibility vary greatly between high and low inflow conditions [55,56], and therefore the behavior of wind power during these periods is extremely important in determining its economic and social benefits.

The widespread weakly positive WD-CUI correlations (Fig 3) suggest that most wind farm sites would have some moderating effect on the climate related variability in provincial electricity supply. However, poorly or negatively correlated sites, such as those in the northern BC coast and the Alaskan panhandle, would provide an even greater moderating influence by having greater than average generation in low inflow years and reduced generation in wet years.

The ability of wind power to address energy deficiency in low water years may be of particular interest to BC because strategically targeted wind power development may help to avoid the impacts of hydroelectric overdevelopment and energy surplus in average and wet years. The North Coast region, including Haida Gwaii and smaller areas of southern and northwestern BC and northern Washington, all had significantly increased wind densities in the lowest inflow years (Fig 4). Wind density totals in these years were larger than the median by as much as 60%, making the value of wind farms in these areas potentially much greater than metrics based solely on annual averages would indicate. Of particular interest are the areas of northern Vancouver Island and the inshore channel off of Haida Gwaii where winds were strong and increasing, and where many wind farms have already been proposed. Of BC’s four existing wind farms only Cape Scott, at the northern tip of Vancouver Island, exhibited significant positive anomalies during low-flow years (Fig 4), with an average increase in WD of nearly 43% for the three-year case, and 26% for the lowest five years. While the three remaining wind farms in the northeastern Peace Region are not likely to have substantially reduced or elevated output during the driest years, they are still likely to produce more energy than an equivalent hydroelectric project, which would likely have much lower production in low inflow years.

Wind speed behavior during high inflow periods should also be considered when selecting wind farm locations. Due to low or negative electricity prices and a reduced ability to manage reservoir levels because of the non-dispatchable nature of wind power, wind generation during high water periods can be of very little value or even detrimental to the operation of the electricity system [13,18,19]. Wind farms that have reduced generation during high water years are therefore preferable to otherwise similar sites with consistent or increased production.

It should also be noted that while the geographic pattern of WD anomalies was similar for the three and five lowest inflow years, the number and magnitude of significant anomalies were lower in the five-year case. That the WD anomalies were generally larger and more numerous when only considering the three driest years suggests that the drivers of the increases or decreases in WD, such as a shift in the storm track, appear to be more consistent for the absolute driest years, rather than for low precipitation years in general. In particular, the almost complete disappearance of significant anomalies in the Far North, Northern Interior, and South Coast in the five-year low inflow case indicates that the wind speed behaviour in these regions is much less persistent across all low-flow years than in other regions with more consistent anomalies, such as the North Coast.

All significant wind density anomalies for the three and five highest inflow years were positive (Fig 5), but were less geographically consistent between the two cases than were the low inflow results. In BC, anomalies in the three-year case were seen in central BC and the Peace region, but in the five-year case anomalies were located mainly to the south, along the Coastal and Rocky Mountain ranges. While the three- and five-year anomaly patterns differed in BC, they were very similar for Washington and Oregon, which exhibited widespread positive anomalies. Interestingly, the WD anomalies in the Peace region, where three of BC’s existing wind farms are located, were larger than the CUI increase for the three highest inflow years, when compared to average years. In contrast, the coastal regions, where the majority of the other potential sites are located, showed no increase in wind density during high inflow years (Fig 5), indicating that wind farms at these locations would have less of an impact on system flexibility in the wettest years.

The different spatial patterns in the three- and five-year high flow anomalies may indicate that the relationships between inflows and WD are less consistent in the wettest years than the driest. This inconsistency, along with the greater importance of understanding WD patterns in low water years to satisfy self-sufficiency requirements, means that high flow anomalies are unlikely to play as large a role in wind farm site selection in BC.

However, the WD anomalies for Washington State and Oregon were more consistent and are therefore likely common to high water years in general. The large positive anomalies found in the US Coast and US Interior, and the rapid wind power development occurring in this region, could result in even larger energy surpluses in high water years, rather than the moderating effect that diversification is meant to bring. Because of the tightly integrated nature of the PNW energy system, such an increase in non-dispatchable generation, when reservoir capacity is already limited, is already having consequences for the electricity market in both Canada and the US. The most significant effect is the decline in electricity prices, particularly during the freshet [57], and therefore a further reduction in the value of wind power and other non-dispatchable sources, such as run-of-river, with increased generation in high inflow years.

### Seasonal Variability

Along with interannual variability, BC inflows undergo large seasonal variations. For example, the average monthly CUI during the spring freshet (May through July) is more than 7 times larger than an average winter month (December through March). Increased wind power during the low inflow winter months can increase system flexibility, and can help to meet higher energy demands caused by increased heating requirements [58]. Electricity prices are also typically highest during the summer and winter [55], making increased energy exports or decreased imports during these periods more profitable.

The largest WD anomalies found during both the winter and freshet time periods were located along the Pacific coast (Fig 6), with decreasing anomaly magnitudes found inland. The large anomalies on the coast appear to reflect the influence of strong winter storms which bring high wind speeds. Relatively lower wind speeds occur during the remainder of the year, including the freshet period when maximum inflow is associated with spring and summer snow melt. Inland areas are also affected by the stronger winds brought by the winter storm tracks but to a much lesser degree, resulting in small positive anomalies in winter and negative anomalies during the freshet, and more consistent wind speeds year-round.

The large decrease in wind speeds and wind densities during the spring freshet for both the Northern Interior and coastal regions (Fig 6) is another major advantage for wind power over expanding hydroelectric generation. The majority of new generation in BC in recent decades has come in the form of run-of-river, which has no storage capacity and experiences a large increase in generation during the spring freshet. “Additional energy during the freshet (May through July) has limited value” [59] because BC Hydro’s reservoirs have limited storage capacity due to high inflows, and because energy prices are typically lower or negative because of surplus hydroelectric generation throughout the PNW [18,19].

### Applicability to Other Regions

While the PNW is an ideal case study area, the proposed methodology to improve the site selection of wind farms by considering the relative long-term variability of wind speeds and hydroelectric reservoir inflows should be beneficial for many energy systems worldwide. A number of factors will affect whether multi-decadal variability could be an important factor in wind farm site selection, including the nature of climate variation, the existing potential for wind power generation, and the heterogeneity within the energy system.

First, the electricity system must contain hydroelectric reservoirs with long-term inflow variability that is large enough to affect system operations or market prices. Such inflow variability could be caused by regular climate cycles (e.g. the Pacific Decadal Oscillation (PDO), North Atlantic Oscillation (NAO), El Niño Southern Oscillation (ENSO)), seasonal rainfall (e.g. monsoon), or snowmelt conditions (e.g. spring freshet). The potential value of poorly correlated wind resources will increase with the magnitude of the inflow variability, and with the importance of hydroelectric generation within the energy system.

Second, there must be substantial wind power potential within the system relative to the total system size or the hydroelectric reservoir capacity. Wind power must have noticeable effects on market prices or reservoir levels for the benefits of increased/decreased wind power during dry/wet periods to outweigh the value of increased total generation at alternative sites.

Third, the wind resource must be sufficiently heterogeneous for there to be regional variations in long-term wind speed variability between potential wind farm sites. In the PNW the long-term variability of both wind speeds [26,27,33,51] and reservoir inflows [52–54] are largely controlled by geographic positioning and local responses to large-scale climate cycles such as ENSO and PDO. As more than 80% of BC Hydro’s hydroelectric generation is produced in the Peace (Northern Interior) and Columbia (Southern Interior) basins (BC Hydro, 2011), CUI variability is largely determined by the streamflow responses in these regions. In contrast, location is the largest determinant of site-specific wind speed behavior [37], which varies widely in the PNW due to the large latitudinal range that covers several climatic regimes and encompasses coastal, mountain, and inland sites with highly complex local topography. The large variability in wind behavior between regions increases the chance that there will be sites with beneficial wind timing and strong wind resources.

While market conditions will determine the nature of the benefits accrued from beneficial wind timing, poorly correlated wind speeds and reservoir inflows can be beneficial in both regulated and deregulated markets. In regulated, or partially regulated, markets, such as BC, wind farms that better complement long-term reservoir inflows can increase energy security and system flexibility, and lower energy costs, which can be passed on to consumers. Similarly, in deregulated markets, building wind farms that reliably generate electricity when prices are higher will increase profitability and decrease market volatility by moderating supply fluctuations.

Some prime examples of where this methodology could be beneficial include New Zealand, Norway, and the Canadian province of Quebec. Hydroelectricity provides the majority of electricity generation in all three jurisdictions, ranging from 50–60% in New Zealand [60] to greater than 95% in Norway and Quebec[61,62]. All three areas also have rapidly growing wind power components [60–62] and the geographic and topographic range to provide a heterogeneous wind resource.

### Caveats

This study suggests that 30-year relationships between wind speed and reservoir inflow behavior can be examined together to improve wind power site selection. However, there are some limitations to interpreting these results that are based largely on using reanalysis data products to determine wind speed behavior. First, meteorological station coverage in BC is very sparse, with even fewer radiosonde locations, forcing NARR to rely heavily on extrapolating down from modeled higher elevation and boundary winds. Previous studies using reanalysis data have calculated surface wind speed trends that are often inconsistent with those seen in observational data [31,63]. Some of the disagreement at the surface may be due to the potentially large effects of local terrain and increasing surface roughness [36] that would not be captured by a reanalysis. However, comparisons at wind turbine hub height have demonstrated greater agreement [35,36,64]. Further work in the areas identified by this study could examine trends at both 10 m and hub height levels.

Second, the length of the NARR data series (30 years) limits our ability to examine longer, historical trends in wind variability, and can increase the possibility that multi-decadal oscillations, such as the PDO, influence the trends. Other work from the Pacific Coast has suggested slight differences in wind speed intensity trends (less significant compared to NARR) when a longer, 60-year time series from the NCAR/NCEP reanalysis is used [50]. Longer term studies have also documented decadal variability in the intensity of Pacific winter storm tracks [49,65], which could influence long-term behaviour of both wind speed and hydrological conditions in the study area. Furthermore, enhanced negative correlations between the North Pacific Gyre Oscillation and winter wind intensity off Vancouver Island are observed when using the longer NCEP time series, suggesting that observed trends may be part of longer-scale, oscillatory climate patterns.

Although our study did not attempt to analyze relationships between climate indices, wind speeds, and hydrological data, we recognize that a longer time series of data could influence the analysis. However, although longer in duration, the NCEP reanalysis has a coarser resolution (2.5°x2.5°, 6-hourly for NCEP; 0.3°x0.3°, 3-hourly for NARR), which tends to reduce the correspondence between NCEP winds and local observed winds, particularly over the topographically complex terrain of British Columbia [66]. As a result, we rely on the 30-year time series of the NARR data.

Another potential limitation of the NARR dataset is that it does not capture the localized variability caused by the highly complex topography of the PNW. The results of this study can therefore only identify regions of interest for further study rather than specific ideal wind farm locations. Correlations between reanalysis wind fields and surface observations are likely to be stronger for coastal sites than for interior sites because of their closer proximity to the climatic conditions over the Pacific Ocean, which are the main drivers of interannual wind speed variation in BC [66]. The stronger correlations between reanalysis and surface observation for coastal sites provide some additional confidence in our identification of northern Vancouver Island, Haida Gwaii, and the North Coast as regions with the most beneficial wind timing for supplementing existing hydropower.

Third, Curry et al. (2012) [66] also showed that downscaling wind speeds from regional predictors, such as the pressure gradient and relative vorticity, as occurs during the production of reanalysis datasets, is much better at capturing local characteristics of interannual variability than monthly, or seasonal variability. As the annual cycle is the dominant mode of variability for monthly and seasonal wind speeds and streamflow, analysis on this time scale simply relates their respective annual cycles, rather than specific local behavior. This lack of local information in the monthly and seasonal data is demonstrated here by the highly uniform winter and freshet WD anomalies. Despite these caveats and limitations, NARR’s limited ability to represent detailed topographic variation in wind speeds at monthly and seasonal time scales should not diminish its usefulness in identifying potential wind farm regions. The CUI-WD relationship is most important on an interannual time scale, for which reanalysis has been shown to be able to distinguish local characteristics [66].

Finally, our study represents a historical analysis of trends and wind-hydrology relationships, but the nature of these relationships could change into the future. Some modeling studies have suggested that the Pacific storm track may shift poleward with global warming [47]. While a northward shifting storm track could result in more storms and, hence, more intense winter wind speeds and higher freshet inflows, it is also likely that warmer temperatures associated with global warming could contribute to changes in the timing of the spring freshet, through earlier snowmelt [67–69]. These changes could reduce the future negative correlations found in this study.

Nevertheless, our analysis provides a means of identifying regions where more detailed analysis with observational data should be prioritized by focusing on areas with low or negative WD-CUI correlations, positive WD anomalies during winter and low inflow years, and negative WD anomalies during the freshet and high inflow years. These areas include northern Vancouver Island, Haida Gwaii, and the broader North Coast region.

## Conclusions

The province of British Columbia (BC) relies heavily on hydroelectric generation, which experiences large fluctuations in generation potential related to interannual and seasonal fluctuations in reservoir inflows. Electricity generation in low flow years is therefore particularly valuable, when hydroelectric generating potential can decline substantially. Using the wind speed data from the North American Regional Reanalysis (NARR) dataset between 1979 and 2010, this study suggests that northern Vancouver Island, Haida Gwaii, and the broader North Coast region all contain sites with strong, broadly increasing winds and negative correlations between annual wind density (WD) and cumulative usable inflows (CUI) estimated by BC Hydro. As such, wind farm sites in these regions could play a particularly useful role in meeting energy generation requirements in the lowest water years and help to moderate the variability in hydroelectric generation.

With a few exceptions (e.g., Columbia River Gorge), wind densities throughout most of the PNW are also significantly higher during the winter and significantly lower during the freshet, when compared to all other months of the year. The Pacific coast again has the most beneficial seasonal timing, with the largest wind density anomalies during both winter and the freshet, along with the western portions of the Rocky Mountains. Widespread decreases in wind density during freshet when system flexibility is low, and increases during winter when capacity demands are highest, indicate that wind power located nearly anywhere in the province would have somewhat beneficial seasonal timing.

While this study has taken a first step in identifying regions with beneficial wind density timing relative to CUI, several additional steps are needed to verify these results. First, this study has identified several interesting candidate regions in BC that should be investigated further by comparing NARR and observational data. In addition to comparisons with existing observational data, there is a need for new meteorological stations that will allow for the testing of the accuracy of NARR’s wind fields and the heterogeneity of the WD-CUI relationships. The inclusion of both surface and turbine height anemometers at these sites would be particularly useful in assessing NARR’s ability to differentiate between surface and boundary layer winds, especially where station data is lacking. Also, wind farms are often located in topographically distinct areas, such as coastlines and ridges. Determining how the wind density behavior at these types of sites compares to the regional results could be important in identifying other regions with beneficial wind timing.

A second step would be to quantify the economic value of developing wind projects in regions with beneficial wind speed variability. Many considerations are included when evaluating potential wind farm sites that determine the cost of energy, such as construction, maintenance, and transmission costs, and the predicted energy output over the turbine's lifespan [70,71]. For the wind density-inflow relationship to be included in this process the relative value of beneficial interannual and seasonal wind density timing compared to these other criteria must first be established. This will be a complex and ongoing process as it depends not only on the variability in electricity prices related to inflows and reservoir levels, but also on more difficult to quantify factors such as system flexibility and the role of wind power in meeting BC's self-imposed self-sufficiency requirement.

## Supporting Information

S1 Table Percent of median annual and seasonal cumulative usable inflow (CUI) totals for British Columbia. Numbers in parentheses are the annual rankings. All totals are based on the water year of October through September, referenced by the calendar year containing October to December. The freshet season is defined as May-July, and the winter season is defined as December-March.
(DOCX)
