# Supplementary material for: The Impacts of Wind Speed Trends and 30-Year Variability in Relation to Hydroelectric Reservoir Inflows on Wind Power in the Pacific Northwest
Source: PLoS One. 2015 Aug 13;10(8):e0135730. doi: 10.1371/journal.pone.0135730 (PMC4535905; doi:10.1371/journal.pone.0135730)
Supplement: S1 Table — Numbers in parentheses are the annual rankings. All totals are based on the water year of October through September, referenced by the calendar year containing October to December. The freshet season is defined as May-July, and the winter season is defined as December-March. (DOCX) [file pone.0135730.s001.docx]

**Table S1****. Percent of median annual and seasonal cumulative usable inflow (CUI) totals for British Columbia.** Numbers in parentheses are the annual rankings. All totals are based on the water year of October through September, referenced by the starting calendar year. The freshet season is defined as May-July, and the winter season is defined as December-March.

| **Year** | **Annual** | **Freshet** | **Winter** |
| --- | --- | --- | --- |
| **1979** | 84.52% (31) | 85.71% (31) | 80.97% (28) |
| **1980** | 110.08% (7) | 109.46% (11) | 130.86% (2) |
| **1981** | 106.02% (9) | 112.55% (7) | 90.14% (25) |
| **1982** | 100.70% (15) | 99.32% (18) | 108.19% (7) |
| **1983** | 98.38% (19) | 96.88% (23) | 97.43% (18) |
| **1984** | 89.80% (28) | 95.15% (25) | 73.64% (32) |
| **1985** | 95.05% (22) | 105.29% (14) | 92.75% (23) |
| **1986** | 102.23% (14) | 110.39% (10) | 101.11% (15) |
| **1987** | 98.82% (18) | 105.45% (13) | 86.88% (26) |
| **1988** | 90.91% (26) | 91.83% (28) | 83.73% (27) |
| **1989** | 105.26% (10) | 112.27% (8) | 102.34% (13) |
| **1990** | 110.23% (6) | 109.33% (12) | 108.23% (6) |
| **1991** | 103.71% (11) | 103.89% (15) | 121.42% (4) |
| **1992** | 89.40% (29) | 87.71% (30) | 79.79% (29) |
| **1993** | 96.67% (20) | 99.01% (20) | 100.75% (16) |
| **1994** | 94.89% (23) | 97.48% (21) | 106.66% (8) |
| **1995** | 119.94% (2) | 121.08% (4) | 123.77% (3) |
| **1996** | 120.37% (1) | 128.81% (2) | 106.64% (9) |
| **1997** | 100.56% (16) | 96.40% (24) | 110.79% (5) |
| **1998** | 112.11% (4) | 116.84% (6) | 106.26% (10) |
| **1999** | 99.44% (17) | 99.66% (17) | 95.15% (20) |
| **2000** | 90.28% (27) | 93.17% (26) | 77.63% (31) |
| **2001** | 109.58% (8) | 126.04% (3) | 94.20% (21) |
| **2002** | 94.38% (24) | 97.34% (22) | 101.97% (14) |
| **2003** | 96.61% (21) | 88.04% (29) | 96.76% (19) |
| **2004** | 103.48% (12) | 100.34% (16) | 132.67% (1) |
| **2005** | 94.12% (25) | 99.27% (19) | 105.83% (11) |
| **2006** | 112.33% (3) | 130.35% (1) | 105.13% (12) |
| **2007** | 102.28% (13) | 111.28% (9) | 93.44% (22) |
| **2008** | 87.32% (30) | 92.63% (27) | 77.77% (30) |
| **2009** | 84.04% (32) | 82.72% (32) | 91.34% (24) |
| **2010** | 110.78% (5) | 120.68% (5) | 99.25% (17) |
